# Extended-Spectrum Beta-Lactamase Escherichia coli-Associated Acute Cholangitis: Uncommon Patient Characteristics and Clinical Implications

**DOI:** 10.7759/cureus.54533

**Published:** 2024-02-20

**Authors:** Somin Lee, Abeer Qasim, Ahmed Alemam, Misbahuddin Khaja, Anil Dev

**Affiliations:** 1 Internal Medicine, BronxCare Health System, Icahn School of Medicine at Mount Sinai, Bronx, USA; 2 Internal Medicine, BronxCare Health System, Bronx, USA; 3 Gastroenterology, BronxCare Health System, Bronx, USA

**Keywords:** extended-spectrum beta-lactamase (esbl), escherichia coli (e. coli), beta-lactamase-producing bacteria, biliary tract infection, acute cholangitis

## Abstract

Acute cholangitis is a potentially life-threatening condition caused by an infection of the biliary tract resulting from biliary obstruction. This case report highlights an unusual presentation of acute cholangitis in an elderly patient characterized by the presence of extended-spectrum beta-lactamase-producing *Escherichia coli.* We aim to emphasize the significance of recognizing diverse clinical manifestations in the elderly population to enhance timely diagnosis and appropriate management. The case highlights the importance of better understanding patient risk factors for potential causative organisms and their susceptibility to selecting proper antibiotics and improving clinical outcomes.

## Introduction

Acute cholangitis is defined as an infection of the biliary tract, primarily a consequence of biliary tract obstruction. From a benign etiology of bile stone and anatomical strictures to malignancy or previous biliary intervention, multiple processes can result in biliary obstruction and subsequent cholangitis [1]. The most frequent cause of biliary obstruction is choledocholithiasis. Other factors contributing to this condition include benign or malignant strictures in the biliary ducts, pancreatic cancer, ampullary adenoma or cancer, tumors in the porta hepatis, parasitic infections such as *Clonorchis sinensis* and *Fasciola hepatica*, as well as infestations by roundworms such as *Ascaris lumbricoides* and tapeworms such as *Taenia saginata*. Additionally, biliary sludge deposits resulting from the obstruction of biliary stents, gallstone impaction in the gallbladder neck or cystic duct causing compression of the common bile or common hepatic duct (known as Mirizzi syndrome), peri-ampullary diverticulum of the duodenum leading to biliary obstruction (known as Lemmel syndrome), and acquired immunodeficiency syndrome can also contribute to biliary obstruction. Due to the possibility of rapid deterioration induced by sepsis, acute cholangitis is a potentially life-threatening situation, with a 30-day all-cause mortality rate ranging from 2.4% to 8.4%, dependent on the severity of cholangitis [2]. Patients with bacteremia are at a higher risk of experiencing complications, including acute renal failure and septic shock [3]. Early detection and efficient treatment are essential to mitigate the risk of complications and mortality. Critical components of acute cholangitis treatment involve biliary drainage and antibiotics [4]. The mortality associated with acute cholangitis escalates when antibiotic therapy is ineffective [3,5]. Improved clinical outcomes can be achieved by selecting appropriate antibiotics based on a thorough understanding of the patient’s risk factors and the susceptibility profiles of potential causative organisms [3]. Here, we present a case of acute cholangitis and bacteremia from an unexpected patient profile with extended-spectrum beta-lactamase (ESBL)-producing *Escherichia coli*.

## Case presentation

A 76-year-old female presented to the emergency department with generalized weakness, fever, and chills for three days. The patient stated she also had abdominal pain for three days, along with multiple episodes of nausea and vomiting. Her past medical history was significant for severe aortic stenosis, hypertension, diabetes mellitus, thyroid disorder, depression, and gastric ulcer. The patient denied any previous biliary intervention or biliary disease. Her only surgical history was transcatheter aortic valve replacement for her aortic stenosis three years ago. The patient denied any recent hospitalization or antibiotics use. She denied any toxic habits including illicit drug abuse. The family history was noncontributory. At presentation, she was tachycardic at 109 beats per minute, febrile at 103°F, and normotensive at 117/54 mmHg. Physical examination was significant for right upper quadrant tenderness and scleral icterus. Laboratory findings were significant for leukocytosis 21.5 k/µL, lactic acidosis 1.8 mmol/L, conjugated hyperbilirubinemia (total bilirubin 3.5 mg/dL, direct bilirubin 3.1 mg/dL), transaminitis alkaline phosphatase 175 U/L, aspartate transaminase 274 U/L, and alanine aminotransferase 337 U/L, suggestive of obstructive biliary disease and inflammation. The patient’s laboratory findings also revealed an elevated international normalized ratio (INR) of 1.5 and elevated creatinine of 2.0 mg/dL, indicating coagulopathy and acute kidney injury. The rest of the laboratory findings are shown in Table 1.

**Table 1:** Patient’s hospitalization days and corresponding laboratory findings.

Laboratory parameter	Day 1	Day 3	Day 5	Day 10	Reference range
White blood cell count	21.5	15.6	36	12.5	4.8–10.8 k/µL
Hemoglobin	10.6	10.2	9.9	8.0	12.0–16.0 g/dL
Hematocrit	33.8	31.7	31.5	25.7	42.0–51.0%
Mean corpuscular volume	82.8	81.4	81.1	81.6	80.0–96.0 fL
Platelet	279	161	211	588	150–400 k/µL
Sodium	133	129	121	132	135–145 mEq/L
Potassium	4.3	3.3	3.3	4.6	3.5–5.0 mEq/L
Bicarbonate	22	22	19	28	24–30 mEq/L
Chloride	97	89	87	95	98–108 mEq/L
Glucose	165	216	133	111	70–120 mg/dL
Blood urea nitrogen	17	17	11	7	8.0–26.0 mg/dL
Creatinine	2.0	0.8	0.7	0.6	0.5–1.5 mg/dL
Calcium	8.3	7.4	7.6	7.6	8.5–10.5 mg/dL
Albumin	3.7	3.3	3.4	2.6	3.4–4.8 g/dL
Total bilirubin	3.5	1.9	1.6	0.8	0.2–1.2 mg/dL
Direct bilirubin	3.1	1.3	1.1	0.4	0.0–0.3 mg/dL
Alkaline phosphatase	175	141	147	105	53–128 U/L
Aspartate transaminase	274	36	36	22	9–48 U/L
Alanine aminotransferase	337	101	63	21	5–40 U/L
Total protein	7.6	6.7	6.6	6.6	6.0–8.5 g/dL
International normalized ratio	1.5	1.12	1.07	1.11	0.85–1.14
Lipase	28	1,495	123	195	*24–151 U/L*
Lactic acid	1.8	n/a	0.9	0.6	0.5–1.6 mmol/L

**Figure 1:**
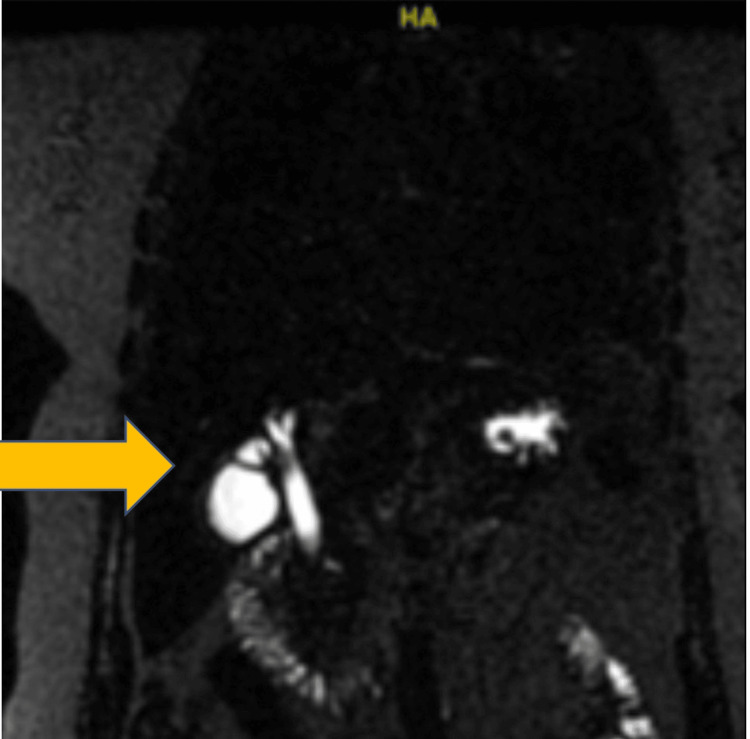
Magnetic resonance cholangiopancreatography showing a filling defect consistent with biliary obstruction.

She underwent an ultrasound of the abdomen, which showed cholelithiasis, a thickened gallbladder wall measuring 3.2 mm, no pericholecystic fluid, negative Murphy’s sign, and no intrahepatic or extrahepatic biliary dilation. The common bile duct (CBD) within normal limits given the patient’s age measured 6.2 mm, despite the initial ultrasound revealing no notable signs of acute obstructive biliary disease. Her clinical presentation of fever, right upper quadrant abdominal pain, jaundice, and laboratory findings were highly suggestive of acute cholangitis and choledocholithiasis. The patient was admitted to the hospital with a working diagnosis of acute cholangitis. She was started empirically on piperacillin-tazobactam and vancomycin. On day two of admission, a subsequent magnetic resonance cholangiopancreatography study showed a 6 mm filling defect within the mid to distal duct, a biliary obstructive finding consistent with choledocholithiasis (Figure 1). Small gallstones were noted within the gallbladder without gallbladder distension or wall edema. The CBD measured 7 to 8 mm.

**Figure 2:**
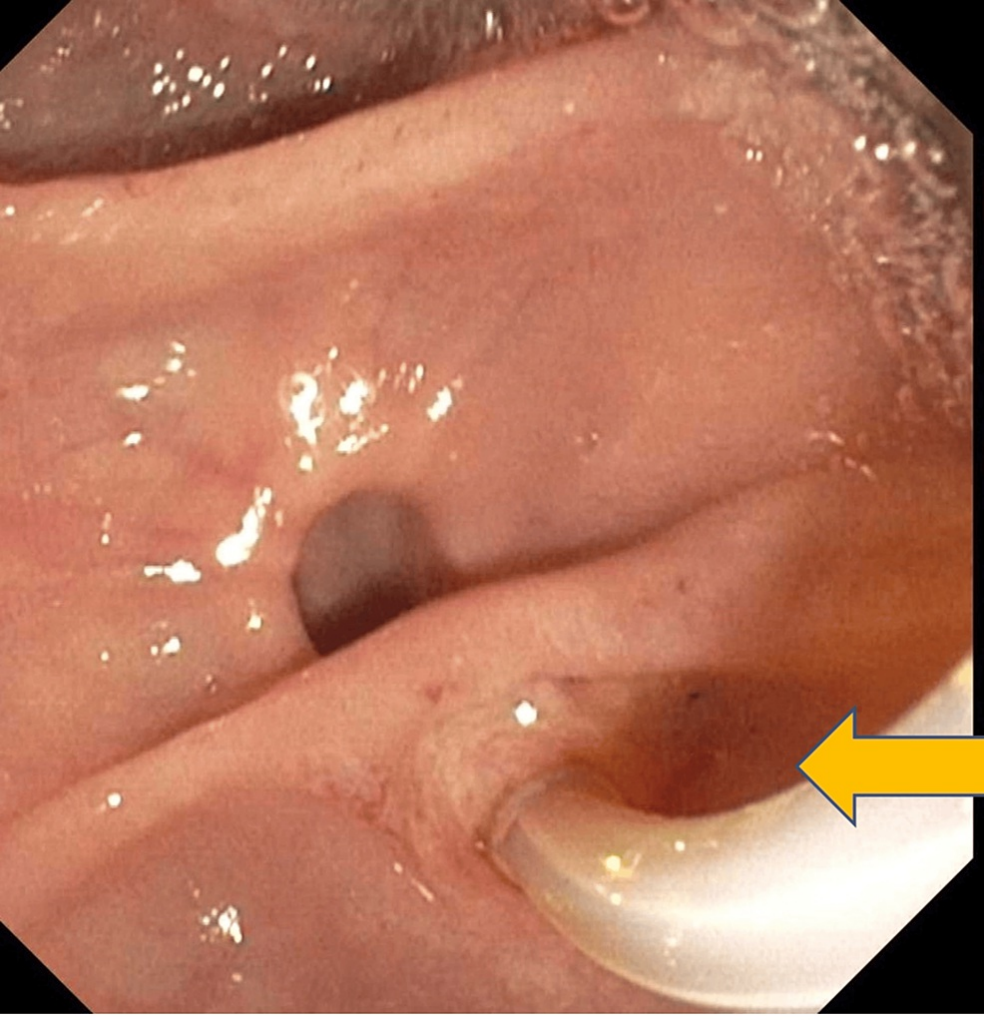
Endoscopic retrograde cholangiopancreatography done to relieve the biliary obstruction.

**Figure 3:**
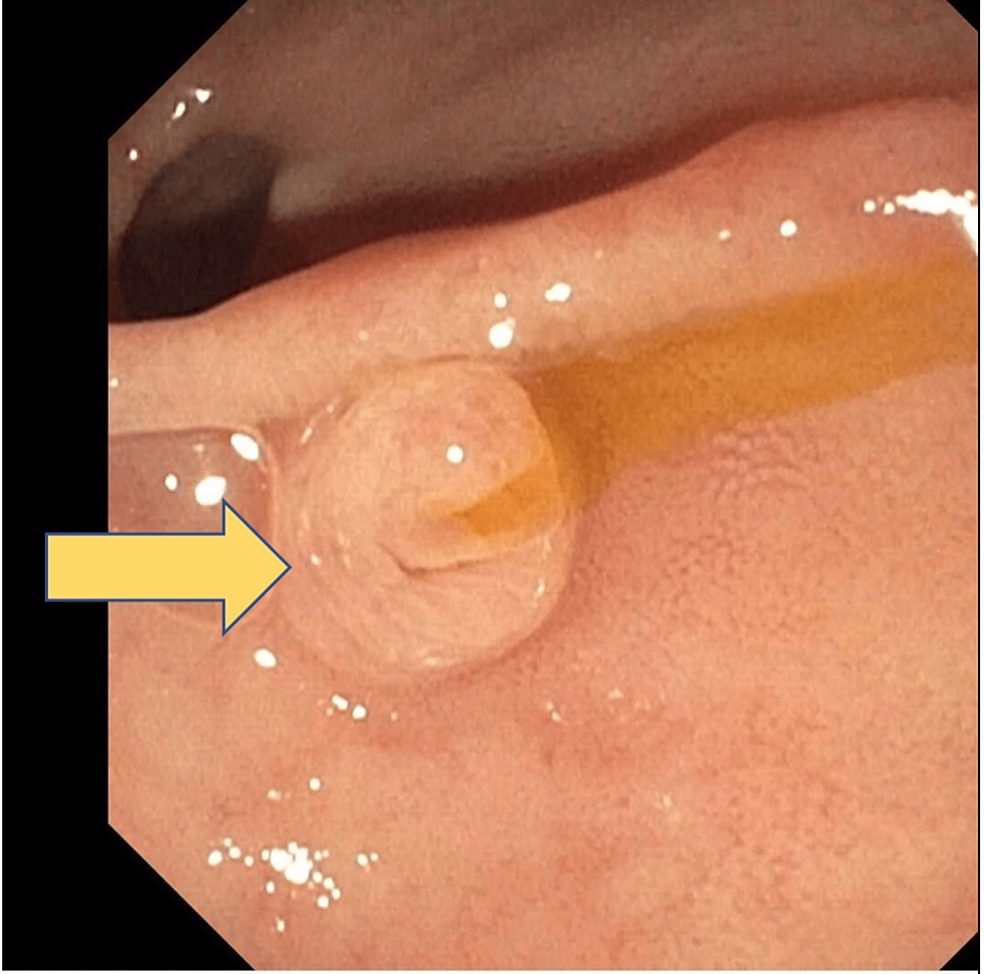
Biliary stent placement as shown by the arrow.

The patient underwent urgent endoscopic retrograde cholangiopancreatography (ERCP) to relieve the biliary obstruction (Figure 2). The contrast was injected. Filling defects were noted on the middle third of the CBD, consistent with stone obstruction. A 7 Fr by 7 cm plastic stent was placed into the CBD (Figure 3). Bile flowed through the stent, and adequate drainage of bile was achieved.

Sphincterotomy was not performed, and stone removal was not attempted, given the condition of severe cholangitis and elevated INR. The patient’s hyperbilirubinemia and transaminitis improved with prompt stent placement and biliary drainage. However, her post-ERCP course was complicated by pancreatitis. Serum lipase trended up to 1,495 U/L. The hospital course noted worsening leukocytosis (36 k/μL) and altered mental status. Patient care was transitioned to critical care. The initial blood culture from the presentation grew ESBL *E. coli*. Antibiotics were switched to meropenem from Zosyn and vancomycin, tailored to the culture report to cover ESBL-resistant *E. coli*. Although her bile culture from ERCP drainage was not sent, given the patient’s clinical course and no other Gram-negative infectious source, the bacteria most likely spread from biliary tract obstruction to the bloodstream. With the start of meropenem, the patient’s leukocytosis resolved, with subsequent negative repeat blood culture confirming the clearing of bacteremia. Transaminitis and elevated lipase resolved to normal limits. Along with the improvement in her clinical course, symptoms of abdominal pain, nausea, and vomiting resolved. The patient was scheduled for elective cholecystectomy, followed by an ERCP in four weeks for stent removal and stone extraction.

## Discussion

Acute cholangitis is a severe and fatal infection if not properly diagnosed and treated. Classic clinical presentation of Charcot’s triad, abdominal pain, fever, and jaundice occurs in 60%-70% of patients with acute cholangitis. The Charcot’s triad has been shown to have high specificity (95.9%) but low sensitivity (26.4%) [1]. In international practice, Tokyo guidelines have been demonstrated to provide an accurate diagnosis in 90% of cases [6,7]. The addition of laboratory and imaging findings consistent with biliary obstruction is included in the guidelines due to the varied presentation of this clinical condition. The Tokyo guidelines help in the early diagnosis of acute cholangitis and identifying the severity to determine the urgency and facilitate early treatment. Key management of acute cholangitis is appropriate resuscitation in case of organ failure and shock, initiation of broad-spectrum antibiotics, and definitive biliary decompression, such as ERCP-guided endoscopic transpapillary biliary drainage, percutaneous transhepatic cholangiography, or surgical drainage. It is recommended to administer broad-spectrum antibiotics until the culture results are available, such as a third-generation cephalosporin or penicillin/beta-lactamase inhibitor [8]. Gram-negative enteric bacteria are the primary pathogens linked to acute cholangitis because biliary tree ascending bacteria from the duodenum are the primary source [9]. *E. coli* and *Klebsiella* spp. have been reported to be the two primary pathogens causing acute cholangitis among a wide variety of bacteria. However, increasing Gram-negative bacteria with ESBL enzymes has recently raised global public health concerns [10]. The increased prevalence of ESBL-producing organisms, attributed to frequent biliary device utilization and prior antibiotic exposure, contributes to inappropriate antibiotic usage and heightened complications and mortality associated with acute cholangitis. It is vital to assess risk factors associated with antibiotic-resistant bacteria for each patient and select proper empiric antibiotics accordingly.

ESBL-producing *E. coli* or *Klebsiella pneumoniae* is predominantly associated with nosocomial acquisition or nursing home exposure. The prevalence of ESBL-producing organisms in community-acquired *E. coli* bacteremia was 4.1%, with 63% attributed to nosocomial infections [11,12]. The incidence of community-acquired ESBL-producing organisms is rising globally [11]. A recent study indicated that certain geographical regions, particularly Asia (specifically Korea and India), exhibit a higher rate of ESBL-producing Gram-negative bacteria in cases of acute cholangitis than other countries [3,9]. In a retrospective study by Goo et al. involving 346 patients, prior biliary intervention (p = 0.016) and nosocomial infection (p = 0.048) were identified as risk factors associated with ESBL or carbapenemase-producing organisms in the bloodstream. Additionally, indwelling biliary devices in the bile were identified as a risk factor (p = 0.024) [3]. Rodríguez-Baño et al. conducted a study on community-acquired ESBL, identifying several potential risk factors for infection with ESBL-producing organisms in the community. These factors included diabetes mellitus, prior fluoroquinolone usage, recurrent urinary tract infections, prior hospital admission, older age, and male gender. While the study encompassed all causes of ESBL-producing *E. coli* infection and was not limited to acute cholangitis, the findings revealed that 76% of cases were associated with urinary tract infections, 22% with asymptomatic bacteriuria, and 2% with acute cholangitis [11].

In our case, the patient was neither a nursing home resident nor acquired nosocomial infections. There was no history of previous biliary intervention, prior fluoroquinolone usage, recurrent urinary tract infections, or prior hospital admission. However, the patient, being elderly and having diabetes mellitus, may have an increased risk of community-acquired ESBL *E. coli* infection. Nevertheless, our patient lacked vital predisposing risk factors documented in the current literature [3,11,12] for ESBL-producing organisms. At the time of presentation, our patient exhibited severe grade 3 acute cholangitis according to TG18/TG13 guidelines, manifesting with more than one organ dysfunction, namely acute kidney injury (elevated creatinine >2.0 mg/dL), and coagulopathy (elevated INR >1.5), indicative of renal and hepatic dysfunction [4,2]. Grade 3 acute cholangitis is characterized by severe cholangitis with sepsis-induced multiple organ dysfunction, requiring urgent biliary drainage to alleviate obstruction and prevent rapid clinical deterioration. Despite biliary drainage, the patient’s condition deteriorated due to the initial inadequate choice of antibiotics. According to the most recent Tokyo guidelines, empirical treatments should include third-generation cephalosporins, piperacillin-tazobactam, or carbapenems, with the choice based on the severity of the illness and local susceptibility data [5,7]. It is essential to emphasize that optimal empirical treatment varies significantly based on local antibiotic sensitivity data, individual risk factors, and the severity of cholangitis. In cases where the clinical condition worsens or severe grade 3 cholangitis is present at the time of presentation, clinicians should broaden antibiotic treatment to cover ESBL-producing organisms, including Gram-positive bacteria such as vancomycin-resistant *Enterococcus *[2], until biliary and blood culture reports are available. Clinicians should be aware that ESBL-producing organisms are prevalent in the community and should not be overlooked when selecting empiric antibiotics.

## Conclusions

Acute cholangitis is a serious condition that requires prompt diagnosis and intervention to prevent severe complications and mortality. While specific, the classic presentation of Charcot’s triad has limited sensitivity, highlighting the importance of utilizing comprehensive diagnostic guidelines such as the Tokyo guidelines for accurate identification of cases. Key management strategies involve resuscitation during organ failure, broad-spectrum antibiotic initiation, and definitive biliary decompression through methods such as ERCP-guided drainage. The prevalence of ESBL-producing bacteria, particularly *E. coli* and *K. pneumoniae*, poses a global challenge, influencing antibiotic choices and patient outcomes. The risk factors for ESBL infection, including prior biliary interventions and nosocomial exposure, need careful consideration when selecting empiric antibiotics. While lacking typical predisposing factors, our patient’s case underscores the evolving landscape of ESBL infections, necessitating a vigilant approach in diverse clinical scenarios. The severity of acute cholangitis, as in our patient with grade 3 disease, emphasizes the urgency of appropriate antibiotic selection based on local susceptibility data. Clinicians must be aware of the rising incidence of community-acquired ESBL-producing organisms and adapt their empirical treatment accordingly, especially when faced with deteriorating clinical conditions. In summary, a tailored and dynamic approach to antibiotic therapy, considering both individual risk factors and disease severity, is crucial in effectively managing acute cholangitis and improving patient outcomes.
